# A Duchenne Muscular Dystrophy Gene Hot Spot Mutation in Dystrophin-Deficient Cavalier King Charles Spaniels Is Amenable to Exon 51 Skipping

**DOI:** 10.1371/journal.pone.0008647

**Published:** 2010-01-13

**Authors:** Gemma L. Walmsley, Virginia Arechavala-Gomeza, Marta Fernandez-Fuente, Margaret M. Burke, Nicole Nagel, Angela Holder, Rachael Stanley, Kate Chandler, Stanley L. Marks, Francesco Muntoni, G. Diane Shelton, Richard J. Piercy

**Affiliations:** 1 Department of Veterinary Clinical Sciences, Royal Veterinary College, London, United Kingdom; 2 Dubowitz Neuromuscular Centre, Institute of Child Health, University College London, United Kingdom; 3 Pathology Laboratory, Harefield Hospital, Royal Brompton & Harefield NHS Foundation Trust, Harefield, Middlesex, United Kingdom; 4 Alphapet Veterinary Clinic, Bognor Regis, West Sussex, United Kingdom; 5 Pathology and Infectious Diseases, Royal Veterinary College, London, United Kingdom; 6 Department of Medicine & Epidemiology, School of Veterinary Medicine, University of California Davis, Davis, California, United States of America; 7 Department of Pathology, School of Medicine, University of California San Diego, La Jolla, California, United States of America; Brigham and Women's Hospital/Harvard Medical School, United States of America

## Abstract

**Background:**

Duchenne muscular dystrophy (DMD), which afflicts 1 in 3500 boys, is one of the most common genetic disorders of children. This fatal degenerative condition is caused by an absence or deficiency of dystrophin in striated muscle. Most affected patients have inherited or spontaneous deletions in the dystrophin gene that disrupt the reading frame resulting in unstable truncated products. For these patients, restoration of the reading frame via antisense oligonucleotide-mediated exon skipping is a promising therapeutic approach. The major DMD deletion “hot spot” is found between exons 45 and 53, and skipping exon 51 in particular is predicted to ameliorate the dystrophic phenotype in the greatest number of patients. Currently the *mdx* mouse is the most widely used animal model of DMD, although its mild phenotype limits its suitability in clinical trials. The Golden Retriever muscular dystrophy (GRMD) model has a severe phenotype, but due to its large size, is expensive to use. Both these models have mutations in regions of the dystrophin gene distant from the commonly mutated DMD “hot spot”.

**Methodology/Principal Findings:**

Here we describe the severe phenotype, histopathological findings, and molecular analysis of Cavalier King Charles Spaniels with dystrophin-deficient muscular dystrophy (CKCS-MD). The dogs harbour a missense mutation in the 5′ donor splice site of exon 50 that results in deletion of exon 50 in mRNA transcripts and a predicted premature truncation of the translated protein. Antisense oligonucleotide-mediated skipping of exon 51 in cultured myoblasts from an affected dog restored the reading frame and protein expression.

**Conclusions/Significance:**

Given the small size of the breed, the amiable temperament and the nature of the mutation, we propose that CKCS-MD is a valuable new model for clinical trials of antisense oligonucleotide-induced exon skipping and other therapeutic approaches for DMD.

## Introduction

The X-linked condition, Duchenne Muscular Dystrophy (DMD), is one of the most common genetic disorders affecting children and young adults. This severe, debilitating and ultimately fatal disease afflicts 1 in 3500 boys. Affected boys are usually wheelchair-bound by their teenage years and death occurs, as a result of respiratory failure or cardiomyopathy, in the late teens or twenties [1]. The disorder results in a progressive degeneration of muscle fibres due to mutations in the gene that encodes the protein dystrophin [2], [3].

Dystrophin is an integral structural component of skeletal and cardiac muscles and connects the contractile apparatus to the sarcolemma. Dystrophin and its associated glycoproteins are believed to provide mechanical stability during muscle contraction; hence its deficiency is postulated to result in fragility of the sarcolemma and subsequently necrosis and, in later stages, replacement of muscle by fibrous tissue. The pathophysiology is not completely understood and alternative pathophysiological mechanisms have been implicated, including alterations in cell signalling, vascular adaptation, inflammatory responses and repair mechanisms [4]–[6].

The dystrophin gene is one of the largest in the human genome (approximately 2.5 million base pairs, encoding 79 exons) and many thousands of mutations are recorded [7]. However, 70% of DMD patients harbour dystrophin gene deletions in a mutation rich area or “hot-spot” in the central genomic region (exons 45–53) [8]–[10]. The dystrophin-deficiency phenotype varies from a severe Duchenne-type to the milder allelic form known as Becker muscular dystrophy (BMD), determined in part by the location of the mutation in relation to the actin and dystroglycan binding domains and, in particular, by the mutation's effect on the reading frame [7]. Mutations that result in premature truncation (for example when a frame shift is induced by an exon deletion) typically result in the severe Duchenne phenotype because no functional dystrophin is produced. Restoration of the reading frame through the use of targeted exon skipping (mediated by antisense oligonucleotides), can generate a functional (but shorter) dystrophin molecule and is a promising area of genetic therapy research [11]–[13]. Indeed, over 90% of human DMD genetic defects are predicted to be amenable to reading frame correction by skipping one or more exons [11], [14]. Skipping one exon in particular (exon 51) is predicted to be suitable for more patients than with any other [11], [12], [15]. Although clinical trials for skipping exon 51 in humans are ongoing, novel antisense chemistries are continuously being developed and methods for refining and optimizing best target selection are urgently needed.

Several animal models are utilised in DMD research. These include the *mdx* mouse, cats with hypertrophic muscular dystrophy, Golden Retriever muscular dystrophy (GRMD) and German Short Haired Pointer muscular dystrophy [12], [16]–[18]. The GRMD model in particular is widely used, as clinically and histopathologically these dogs generally have a severe phenotype that resembles human DMD; their use in trials of stem cell based therapies [19] and in exon-skipping therapy [20] has been encouraging. Unfortunately the Golden Retriever's large size (approximately 25–30 kg) has implications for treatment costs in therapeutic trials; furthermore, handling these large dogs has substantial welfare implications (and high associated costs) due to their debilitating condition. To reduce these issues, the same mutation was bred into a colony of beagles to establish Canine X-linked Muscular Dystrophy in Japan (CXMD_J_) [21] which subsequently has been used to demonstrate efficacy of exon skipping therapy [22].

Each of the animal models used in DMD research has associated benefits and disadvantages, but none harbours a mutation in the region of the dystrophin gene that is most commonly mutated in human DMD. New canine models of DMD with different mutations would therefore significantly compliment research, particularly therapeutic trials. Here we describe a spontaneous canine model of DMD in the Cavalier King Charles Spaniel (CKCS), a toy breed, specifically bred for its small size (5–8 kg) and amiable temperament. In this manuscript we report the clinical, pathological and molecular characterisation of the model and investigate the suitability of exon skipping-mediated therapy.

## Results

### Dogs with CKCS-MD Have a Severe Dystrophic Phenotype Similar to DMD

The index case was a 10-month-old male neutered client-owned CKCS from the United Kingdom that was presented to the Royal Veterinary College for investigation of a generalised neuromuscular disorder. The dog had been in the owners' possession from 2 months of age and the puppy had always been of small stature, poorly muscled and exercise intolerant; the main presenting complaint, however, was a 3 month progressive history of dysphagia.

**Figure 1:**
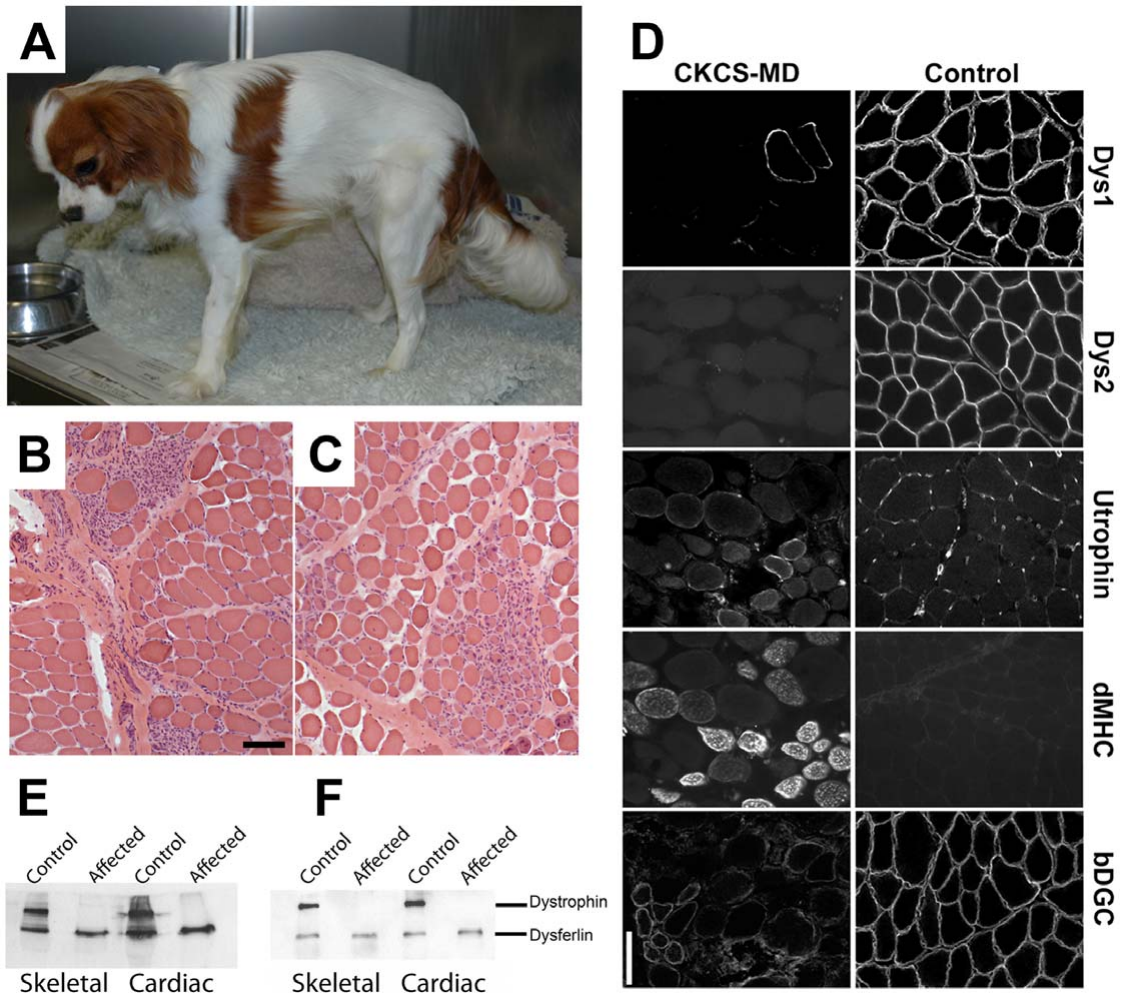
CKCS-MD phenotype. (A) Photograph of the index case at 10 months of age. Note the generalised muscle atrophy and the low head carriage, indicative of paresis. (B and C) Representative cryosections of the vastus lateralis muscle from a dystrophic CKCS dog (dog 2) showing excessive variability in myofiber size and typical areas of degeneration (necrosis and phagocytosis, B) and regeneration (small fibers with prominent vesicular central nuclei, C). H&E stain, bar = 100 µm for both B and C. (D) Immunohistochemistry of CKCS-MD skeletal muscle (left column) compared with control dog muscle (right column), labeled with antibodies to the dystrophin rod (Dys1) and carboxy (Dys2) domains, utrophin, developmental myosin heavy chain (dMHC) and β-dystroglycan (bDGC). Note the absence of expression of dystrophin (with the exception of occasional revertant fibers), redistribution of utrophin to the sarcolemma in both regenerating and non-regenerating fibers, expression of developmental myosin heavy chain and reduced/variable expression of β-dystroglycan in the affected dog in comparison with control. Bar = 100 µm for all images. The utrophin and developmental myosin heavy chain labeling is on serial sections. (E and F) Western immunoblot of extracts from skeletal and cardiac muscle from the index case using antibodies to (E) the dystrophin rod (Dys1) and (F) the carboxy (Dys2) domains in comparison with control muscle. Note the total absence of dystrophin in both skeletal and cardiac muscle in the affected dog. The positive bands, detected using an antibody to dysferlin, confirm equal protein loading in each lane.

On examination the dog was tetraparetic with low body condition score (2/9; weight = 4.7 kg) (figure 1A), reduced withdrawal reflexes, macroglossia and restricted jaw movement. Investigations documented a marked elevation in serum creatine kinase (CK) activity (33,695 U/l; normal range 61–394 U/l). Electromyography revealed a myopathic picture with associated spontaneous activity (complex repetitive discharges and pseudomyotonia). Thoracic radiography revealed a normal size and shape to the cardiac silhouette. The dog was diagnosed with dystrophin deficient muscular dystrophy (see below). Clinical signs progressed and euthanasia was performed at the owners' request at 24 months of age due to progressive dysphagia and recurrent aspiration pneumonia.

Two additional young male CKCS from the USA were also diagnosed with dystrophin deficient muscular dystrophy (dogs 2 and 3). Dog 2 was a 6-month-old intact male that was presented to a general veterinarian because of dysphagia and decreased range of jaw motion, first noted by the owner at 2 months of age. A marked elevation in serum CK activity (64,918 U/L) was measured. Dog 3 was an 8-month-old neutered male that was presented to the Veterinary Teaching Hospital, University of California, Davis, with a history of gagging when eating and drinking since acquired as a 6-week-old puppy, combined with exercise intolerance. On physical examination, restricted jaw mobility and macroglossia were noted. The serum CK activity was markedly elevated (58,508 U/L). Thoracic radiography revealed a normal shape and size to the cardiac silhouette and echocardiography revealed very mild mitral and tricuspid regurgitation (believed to be clinically irrelevant) and normal left ventricular function. Electromyography revealed spontaneous repetitive discharges and pseudomyotonia in all muscles examined.

### Histopathology

Open muscle biopsies were obtained from all 3 dogs under general anesthesia. The cranial tibial muscle was sampled for the index case, and the vastus lateralis and triceps muscles for dogs 2 and 3. A dystrophic phenotype was present in all muscle biopsy specimens (figures 1B and 1C). Excessive variability in myofiber size was evident with a pattern of degeneration (multifocal groups of necrotic fibers with variable phagocytosis) and regeneration (multifocal groups of small calibre fibers with a basophilic appearance and of histochemical type 2C). Rare calcified deposits were also observed.

### Cardiac Pathology

The heart from dog 1 was of normal size (weighing 35 g) and was structurally normal with a left-dominant coronary circulation. There was good preservation of myocyte structure with no myofibrillary disarray, myocyte hypertrophy, vacuolation or attenuation. Occasional myocytes in both ventricles showed hypereosinophilia and loss of myofibril structure without associated inflammation (changes regarded as agonal rather than representing intrinsic myocyte pathology). There was no increase in stromal fibrous tissue apart from focal fibrosis in a papillary muscle adjacent to an area of endocardial fibroelastosis. Papillary muscle vessels showed some medial hypertrophy. The above pathological changes (not shown) were regarded as non-specific.

### Evaluation of Dystrophin and Dystrophin Associated Proteins in CKCS-MD

Dystrophin was absent in skeletal muscle from all dogs (detected by immunohistochemistry using antibodies to both the dystrophin rod domain and C terminus; figure 1D) apart from occasional scattered positive revertant fibres (figure 1D). Dystrophin was similarly absent in both skeletal and cardiac muscle of dog 1 by immunoblot (figures 1E and 1F). Abundant expression of developmental myosin heavy chain in muscle from dog 2, confirmed regeneration (figure 1D). Utrophin expression was examined in muscle from dog 2 and found to be over-expressed and relocalised to the sarcolemma in both regenerating and non-regenerating fibres (figure 1D). As in DMD, in muscle from dog 1 there was reduced expression of β-dystroglycan (figure 1D) and γ-sarcoglycan and sarcoplasmic redistribution of neuronal nitric oxide synthase (not shown) [23].

### Molecular Analysis

**Figure 2:**
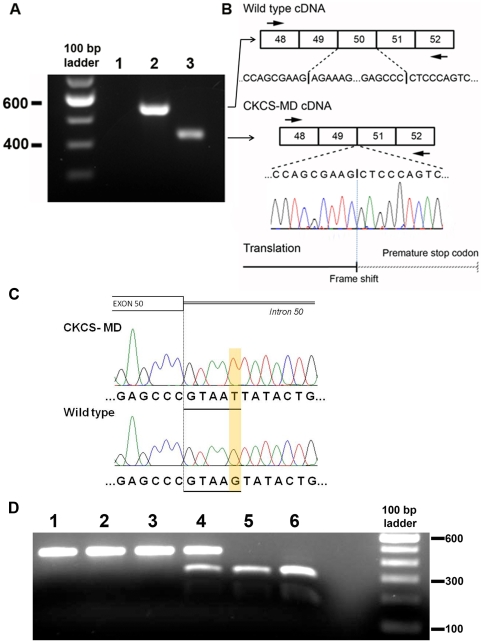
Genotype of CKCS-MD. (A and B) RT-PCR of mRNA extracted from skeletal muscle from a control dog (2) and the index case (3) using primer pair 2–10. Lane 1 is a water control. Note the expected amplicon size of 547 bp in the control but a shorter fragment in the affected dog (A) which upon sequencing was determined to be due to deletion of the 109 bp in exon 50 (B). (C) Direct sequencing of genomic DNA from a control (wild type) dog and from the index case (CKCS-MD) revealed a G-T missense mutation (highlighted) in the 5′ consensus splice site (underlined) in intron 50. (**D**) Restriction fragment length polymorphism (RFLP) assay using restriction enzyme BSTZ17I. The splice site mutation in the 5′ consensus splice site of intron 50 in the index case, dog 2 and dog 3 (lanes 1, 2 and 3 respectively) is identified by the fact that mutant DNA remains undigested (449 bp) whereas wild type DNA is digested into 2 smaller products of 296 and 153 bp. Lane 4 contains restricted PCR products from dog 3's mother, confirming her as a carrier. Lane 5 (dog 3's father) and lane 6 (control dog) contain restricted PCR products that are wild type.

RNA was extracted from frozen skeletal muscle and cDNA synthesised by reverse transcription. PCR primers were used to amplify overlapping regions of dystrophin cDNA of 1000–1500 base pairs in length for comparison of cDNA from the index case and a control dog. No difference in fragment sizes between the affected and control dog PCR products could be appreciated (results not shown). Primers were then designed to amplify via PCRs, overlapping regions of 500–600 base pairs suitable for sequencing, concentrating initially on the central region of the gene. Of these, PCR with one primer pair (2-10F–2-10R (table S1) generated a shorter product from dog 1's cDNA in comparison with cDNA from a control dog (figure 2A) that upon sequencing was determined to be due to a deletion of the 109 bp in exon 50 (figure 2B). The deletion results in a frame shift and a premature stop codon 24 base pairs into exon 51.

Genomic DNA was subsequently used as the template to amplify by PCR, a 635 bp fragment that included exon 50 and intronic splice sites in introns 49 and 50 (primer pair 3-1 (table S1). Sequencing confirmed a splice mutation (G–T) in the 5′ consensus donor splice site within intron 50 in the genomic DNA from dog 1 that was absent in DNA from a control dog and the canine reference sequence (GenBank NC_006621) (figure 2C); the same mutation was subsequently identified by direct sequencing genomic DNA from dog 2.

### Restriction Fragment Length Polymorphism Assay

A restriction fragment length polymorphism (RFLP) assay that differentiates the mutant from the wild type allele using the restriction enzyme BSTZ17I was subsequently used to demonstrate the presence of the mutation in dogs 1, 2 and 3 (figure 2D). The RFLP also identified the carrier status of dog 3's dam and absence of the mutation in dog 3's sire (figure 2D). Screening of an additional 96 unrelated female CKCS from the UK (192 alleles) identified no further heterozygous (carrier) females (all dogs had the wild type sequence) (results not shown).

### Antisense Oligonucleotide Induced Exon-Skipping

**Figure 3:**
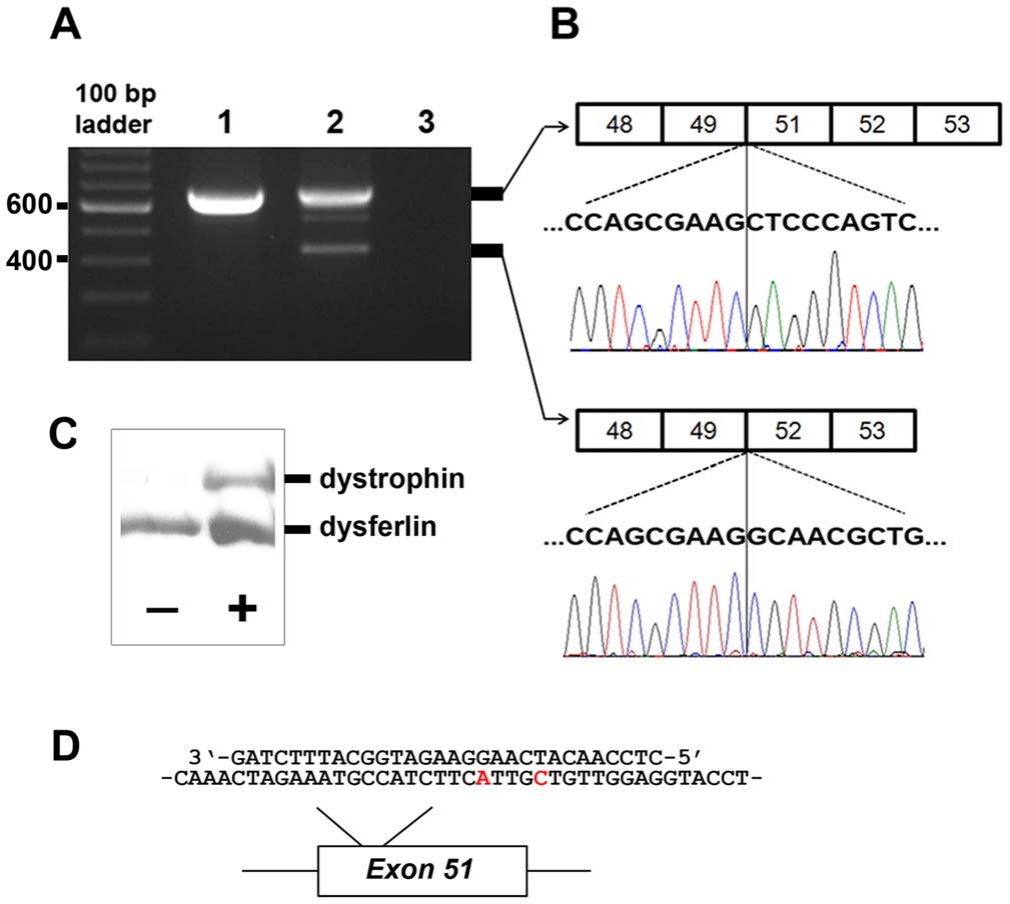
Antisense oligonucleotide mediated skipping of exon 51 restores protein expression. (A) RT-PCR of mRNA extracted from cultured canine myoblasts derived from the index case in the absence (lane 1) or presence (lane 2) of AVI-4658; lane 3 is a water control. Note the 666 bp product of the unskipped cDNA (upper band) consistent with the deletion of exon 50 (caused by the mutation) and the smaller 433 bp product consistent with skipping of exon 51. Sequences were confirmed in the extracted products (B). (C) Western immunoblot of protein extracts from treated (+) and untreated (−) CKCS-MD myoblasts using antibodies to the dystrophin rod (Dys1) and dysferlin (as a loading control) demonstrating re-expression of dystrophin following exon 51 skipping in treated cells. (**D**) Sequence alignment of AVI-4658 (above) with part of the canine exon 51 sequence, demonstrating the mismatches (in red) analogous to the differences between the canine and human sequences.

To demonstrate the potential of antisense oligonucleotide (AO) mediated skipping to correct the reading frame in mutant transcripts from the affected animal, we treated cultured myoblasts from the index case with an exon-internal AO designed to induce skipping of exon 51 in humans and compared mRNA expression with untreated myoblasts [15]. RT-PCR of mRNA extracted from treated and untreated cells revealed the expected sized product (with absence of exon 50), however in the treated cells, a smaller sized product was present, compatible with skipping of exon 51 (figure 3). Direct sequencing of both bands confirmed deletion of exon 50 in the larger band and deletion of both exon 50 and 51 in the smaller band (figure 3). Western blot analysis of the lysates of treated myoblasts, in comparison with untreated cells, revealed restoration of dystrophin protein expression (figure 3).

## Discussion

In this study we sought to determine the genetic basis of dystrophin-deficient muscular dystrophy in 3 CKCS dogs. The novel splice site mutation of the dystrophin gene that we have identified in these affected dogs from the UK and the USA occurs in a region that is most commonly mutated in humans, and gives rise to a single exon deletion (exon 50) in the dystrophin mRNA. This generates a frame shift resulting in a predicted premature truncation of the gene product. Given that all affected animals have a severe phenotype, analogous to DMD in humans, we propose that CKCS with this mutation offer a valuable addition to the existing repertoire of animal models of DMD. Indeed, this new canine model may be preferable, given the nature and site of the mutation and the breed's small size.

Existing models of DMD include the *mdx* mouse, which has a nonsense mutation and premature stop codon within exon 23; however, the clinical and histological phenotype is much less severe than would be expected in a human patient with an equivalent mutation. Indeed, these mice have a near normal life span and mobility, without significant muscle fibrosis or joint contractures. Other dystrophin-null mice generated by 5 N-ethyl-N-nitrosurea mutagenesis [18], [24], [25] are also mildly affected and maintain a normal muscle regenerative capacity when compared with human DMD [17], [18]. Researchers have therefore increased the severity of the murine dystrophin-deficiency phenotype by creating double mutants: either mice lacking both dystrophin and its paralogue, utrophin, or both dystrophin and MyoD (a transcription factor important in muscle development and regeneration) [18], [26]. However, despite the obvious benefits of murine models (including experimental simplicity, small size, familiarity, ease of husbandry and relative low costs), these differences mean that these animals do not faithfully model the clinical situation in DMD: therapeutic trials can be hard to assess in mildly affected *mdx* mice and are difficult to interpret in double-mutant mice [18].

Several larger animal models of dystrophin deficiency have more severe phenotypes, although a variety of limitations remains. The analogous feline condition (Feline Hypertrophic Muscular Dystrophy) [27], [28] though severe, is phenotypically distinct from canine and human dystrophinopathies, being characterised by generalised muscle hypertrophy, exuberant regeneration and marked calcification [29]. In dogs, spontaneously occurring distinct genetic defects leading to dystrophin-deficient muscular dystrophy have been described in the Golden Retriever and German Short Haired Pointer [16], [17], [30], [31] Schatzberg et al. [16] described two male German Short Haired Pointer littermates with a large (>2.4 Mb) deletion of the p21 region of the X-chromosome that included the dystrophin gene and promoters. The phenotype of these cases and progression was only briefly described; however, there was striking heterogeneity–one dog was severely affected with a high serum CK activity but a full sibling was relatively mildly affected and had a correspondingly lower CK activity [16], [32].

The GRMD model is caused by a point mutation in the 3′ splice site of exon 7 (resulting in exon 7's deletion and termination within exon 8) [31]. Several colonies exist worldwide, all descended from the same founder male [31]. Subsequently, the same mutation on a beagle background (Canine X-linked Muscular Dystrophy Japan CXMDj) was established because of the beagle's smaller size (10–15 kg) and the breed's established use as an experimental animal [21]. The GRMD clinical and histopathological phenotype resembles DMD: typically elevated serum CK activity is detectable shortly after birth, weakness is evident within the first few weeks and joint contractures significantly impair movement by 6 months. Death from respiratory or cardiac failure occurs around 18 months of age [32]. Beagles with the same mutation are reported to have a milder phenotype [21], [33]. Similar to the cases we describe in CKCS, macroglossia and dysphagia are prominent features of both GRMD and CXMDj [32], [33]. Dogs with dystrophin-deficient muscular dystrophy (GRMD, CXMDj, CKCS-MD) retain ambulatory function until fairly late in the disease's course, unlike boys with DMD who require wheelchairs relatively early, likely reflecting the relative differences between quadrupedal versus bipedal locomotion when adapting to generalised paresis.

The phenotype in both GRMD and CXMDj is variable–from clinically unaffected dogs [34] to fulminant neonatal disease and early death [32], [33]. Banks and Chamberlain [18] proposed that variation may result from differences in expression of truncated dystrophin transcripts [35] that influence dystrophin expression and its function through the N-terminal actin-binding domain. Heterogeneity is also well documented in DMD and is attributed also to environmental and modifying gene effects [36]. Utrophin expression in particular is believed to be partially protective; the protein is generally relocalised to the sarcolemma from its normal adult position at the neuromuscular junction in DMD patient muscle [37] and its artificial upregulation in *mdx* mice and in GRMD is protective [38], [39]. Utrophin's sarcolemmal expression was demonstrated in the muscle from one of the CKCS dogs in the current study in some non-regenerating fibres.

DMD models should be well characterised and it is essential to consider inherent clinical variability when interpreting the results of therapeutic trials. In this study we report the clinical features of 3 independent client-owned animals that were presented for veterinary investigation. All affected dogs had a similar clinical presentation for dysphagia and macroglossia since an early age. Further, they all had a similar dystrophic phenotype in muscle biopsy specimens. It is noteworthy, that none of the dogs in the current study had either clinical or histopathological signs of cardiac disease, even though dilated cardiomyopathy is a common feature in DMD [40]. In CXMDj myocardial fibrosis occurs later and is milder than in GRMD [41], [42]: one explanation therefore for the absence of evidence of cardiac disease in dogs in the current study may be the length of time required for the genetic abnormality to be manifested in heart as opposed to skeletal muscle in this small breed. Further work, ideally utilizing a colony of CKCS-MD dogs, would enable comprehensive phenotypic characterization of various organ systems, including the heart. Furthermore, if the cardiac muscle changes are found to be comparatively mild in this model, it would provide an opportunity to evaluate factors contributing to this phenotype.

Over 70% of human dystrophin gene mutations are intragenic deletions of varying size [7]. The majority occur in the repetitive central area of the gene (exons 45–53)–designated the major deletion hot spot [8]–[10]–because of the gene's propensity to undergo recombination in this region [43], [44]. For this reason, we chose to concentrate our search for the causative mutation in this region of the canine gene, following an early wider screen of the entire cDNA. In humans, the phenotype depends on the effect of the deletion on the reading frame, with the more severe Duchenne phenotype, typically occurring when there is a frame shift that results in premature truncation [7]. In CKCS-MD, a transition mutation in intron 50 (the 5′ (donor) splice site of exon 50) causes deletion of exon 50 from the dystrophin mRNA, a frame shift and premature truncation in exon 51 and a severe phenotype in the reported dogs. The index case was of unknown pedigree therefore it has not been possible to confirm the relationship between affected dogs. Nonetheless, given that we detected the same mutation in dogs of the same breed from the UK and the USA and considering the narrow genetic pool of the CKCS breed [45], it is most likely that affected dogs are all related to a single founder. The worldwide prevalence of this mutation is currently unknown: as no carriers were identified in 96 female CKCS from the United Kingdom the defect may be present at a low level within the breed in this country.

Observation that frame shift deletions in humans cause DMD, but that sometimes larger, in frame deletions, have a much milder Becker phenotype, prompted investigation of one of the more promising approaches towards genetic therapy: the use of exogenous targeted antisense oligonucleotides to mediate artificial splice defects and induce skipping of additional exons in order to restore the reading frame [11], [12]. Production of these shorter, but likely functional dystrophin molecules occurs naturally in many patients with DMD, and can be demonstrated immunohistochemically, by the presence of revertant muscle fibres [46]–[47] as seen in two of the affected CKCS dogs in this study. Some authors suggest that natural, albeit low level expression of revertant dystrophin in humans may prevent any immune response on dystrophin reexpression induced through gene therapy [18].

Although not all DMD mutations are amenable to antisense therapy–for example large deletions of the dystrophin gene or those affecting the cysteine-rich area–researchers estimate that exon skipping therapy may be applicable in over 90% of DMD patients [11], [14]. In this study, we have demonstrated the application of antisense therapy to induce skipping of exon 51 in CKCS-MD and restore dystrophin expression: the identical approach (skipping the same exon) has the potential to restore the reading frame and produce functional dystrophin and improved clinical signs in more DMD patients than with any other site (up to 13% of DMD patients) [11], [12], [15]. Indeed for the current study, we utilised a human-adapted exon-internal antisense oligonucleotide [15] that is undergoing phase I/II clinical trials in humans (ClinicalTrials.gov NCT00159250 and NCT00844597) [14]. It is noteworthy that the canine and human exon 51 sequences targeted by AVI-4658 differ by 2 nucleotides (figure 3): optimisation of an analogous canine AVI-4658 sequence may further improve skipping efficiency in this model.

AO-mediated exon skipping was demonstrated *in vitro* in GRMD [21] and a recent study demonstrated the efficacy and safety of systemic delivery in the CXMDj model [22]. The genetic defect in GRMD and CXMDj models results in deletion of exon 7; however, the length of adjacent exons is such that skipping of multiple additional exons (6–8 or 6–9) is required to restore the reading frame [22]. These recent trials in CXMDj utilised very high and frequent doses of systemically delivered antisense oligonucleotides; additional work, in particular, improving delivery, efficacy and reducing dose, frequency and the potential for toxicity is very important for similar systemic therapeutic trials in humans. We suggest the CKCS-MD model offers an opportunity to optimise exon-skipping approaches in a more clinically relevant region of the dystrophin gene than in either GRMD or CXMDj. Alternatively, given the established use of beagles as a laboratory animal and application of exon skipping therapies in this breed, it may be advantageous to establish the CKCS-MD mutation in a beagle colony as performed for the GRMD genotype with CXMDj [21].

In summary, we have described the clinical phenotype and revealed the genetic cause of dystrophin deficient muscular dystrophy in a group of CKCS: a deletion in the region of the dystrophin gene that is most commonly mutated in DMD. Current evidence suggests that unlike *mdx* mice and certain other larger animal models of DMD, the severe DMD-like phenotype in these dogs means they may prove useful in trials of antisense-oligonucleotide induced exon skipping and other treatments, such as stem cell therapy [12], [19]. The small size of this breed and its amiable temperament are additional advantages since costs for therapeutic trials, maintenance and care would be substantially lower than in other larger canine models.

## Materials and Methods

All clinical and diagnostic veterinary procedures on animals in this study were performed with informed owner consent by licensed veterinary surgeons.

### Clinical Evaluation

Physical and neurological examinations were performed in a standard manner. Electromyography and muscle biopsy were conducted under general anaesthesia. The former included assessment of tongue and temporal muscles, epaxial muscles, a selection of appendicular muscles (including flexors and extensors at various depths) in both pelvic and thoracic limbs using digital electrodiagnostic equipment (Medelec Synergy, Oxford Instruments). Open muscle biopsy samples were obtained from the cranial tibial muscle (index case at age 10 months) and from the triceps and vastus lateralis muscles in dogs 2 and 3 (aged 6 months and 8 months respectively).

In the index case, immediately post intravenous barbiturate euthanasia at 24 months, samples (approximately 1 g) of skeletal muscle and left ventricle were frozen in cryotubes in dry ice before being stored at −80°C for western immunoblot analysis. The remainder of the heart was fixed in 10% buffered formalin. Additional (approximately 1 g) pieces of skeletal muscle from the affected dog were stored in tissue culture medium (10% foetal calf serum, Dulbecco Modified Eagle's Medium, 10 mM L-glutamine and penicillin-streptomycin) at 4°C for up to 48 hours until culture.

### Muscle Biopsy Samples

Immediately following biopsy, skeletal muscle samples were positioned on cork discs so that the fibres were orientated vertically and frozen in optimal cooling temperature compound (Tissuetek) in isopentane that had been precooled in liquid nitrogen [48]. Samples were cryosectioned at 7 µm (for immunohistochemistry) and 10 µm for other stains and histochemical reactions. Unstained cryosections were stained or reacted with haematoxylin and eosin, modified Gomori trichrome, periodic acid schiff, ATPases at pH 9.8 and 4.3, esterase, NADH-TR, acid phosphatase, alkaline phosphatase, oil red O and SPA-HRPO by standard methods [48].

### Immunohistochemistry

Immunohistochemistry was performed by incubating 7 µm skeletal muscle cryosections with primary antibodies (diluted in 0.2 M PBS, pH 7.2) for 1 hour at room temperature. After 3 washes in PBS, biotinylated goat secondary antibodies (1 in 200; GE Healthcare) were applied for 30 minutes. After a further 3 washes streptavidin Alexa 594 (1 in 2000; Molecular Probes; Invitrogen) was added for 30 minutes. The primary antibodies used were (Novocastra) mouse monoclonal antibodies to the dystrophin rod and carboxy domains (Dys1 (1∶2) and Dys 2 (1∶20), anti-β-dystroglycan (1∶20, 43DAG/8D5), γ-sarcoglycan (1∶30; 35DAG/21B5), utrophin (1∶20; DRP2) and developmental myosin heavy chain (1∶20; NCL-MHCd) and a rabbit polyclonal antibody to nNOS (1∶50; R20; Santa Cruz).

### Heart

The formalin fixed cardiac muscle obtained post-mortem in the index case was analysed grossly and histologically. For the latter, targeted sections were taken from the septum and free walls of both ventricles of a midventricular transverse slice of myocardium. They were processed routinely to paraffin wax and 4 µm sections stained with haematoxylin-eosin for cell morphology and Masson trichrome, Verhoeff elastic-Van Gieson and picrosirius red stains for connective tissue content.

### Western Immunoblot

Western immunoblots using approximately 30 mg of skeletal and cardiac muscle from a control dog and from the index case, and from cultured myoblasts, were performed using antibodies to Dys1 and Dys2 and dysferlin (loading control) (all Novocastra) via a 6% polyacrylamide gel with a 4% stacking gel using routine methods described by Arechevala-Gomeza et al [14].

### Genotyping

Total RNA was extracted from frozen muscle using the RNeasy mini kit (Qiagen) and cDNA was synthesised by reverse transcription (SuperScript III, Invitrogen) with random hexameric primers according to the manufacturer's instructions. Genomic DNA was extracted from EDTA anti-coagulated blood or frozen muscle using the DNeasy mini kit (Qiagen) according to the manufacturer's protocols.

PCR primers designed using Primer3 (version 0.4.0; http://frodo.wi.mit.edu) were based on the published sequence for canine dystrophin mRNA (GenBank NM_001003343) or gDNA (GenBank NC_006621). Initially these were designed to amplify overlapping sections of the dystrophin cDNA each of 1000–1500 base pairs in length (cDNA primers 1-1 to 1-12; table S1) in order to screen for large deletions by comparing product sizes from a control and CKCS-MD affected dog. A further set of PCR primers was designed to amplify 500–600 base pair segments suitable for sequencing (cDNA primers 2-1 to 2-10; table S1). Genomic DNA containing exon 50 was amplified by PCR using primer pair 3-1 (table S1). PCRs were performed using AmpliTaq Gold polymerase (Applied Biosystems) according to the manufacturer's directions. Sequencing was performed using core facilities.

### Restriction Fragment Length Polymorphism Assay

Genomic DNA samples from each of the 3 affected dogs, the parents of dog 3 and from 96 additional female CKCS dogs were examined. The latter samples were extracted from stored frozen whole blood in EDTA derived from CKCSs that had been presented to the Royal Veterinary College for evaluation of various medical and surgical conditions following owner consent. DNA was extracted using the 96 well plate procedure (DNeasy 96 kit, Qiagen) according to the manufacturer's directions. PCR was conducted as above (primer pair 3-2; table S1) and the 449 bp fragment purified as above. Products were incubated with BSTZ17I enzyme (New England Biolabs) according to manufacturer's directions. The mutant sequence was predicted to remain uncut whereas digestion of PCR products from unaffected dogs was predicted to result in 2 smaller bands of 296 and 153 bp. Carrier females were predicted to be identified by the smaller normal allele bands and full sized mutant product.

### Myoblast Culture

Myoblasts were cultured from muscle explants using established techniques. Skeletal muscle from dog 1 was cut into 1 mm cubes with a sterile scalpel blade, placed into the centre of 3.5 cm tissue culture dishes, and maintained in an incubator (37°C and 5% CO_2_) within a single drop of growth medium (DMEM (Dulbecco's Modified Eagle's Medium), 10% FBS (fetal bovine serum), penicillin-streptomycin (1 U/ml and 1 µg/ml, respectively), and 2 mM l-glutamine). After 6 hours, another 2 ml of medium was added and the culture dishes were returned to the incubator for 2 to 3 days, by which time myoblast-like cells were readily visible migrating outward from the tissue. Fresh medium was then added and cultures were maintained for an additional 7 to 10 days until a confluent bed of myoblast-like cells had been obtained. Aliquots were stored frozen until used.

### Transient Transfection Experiments and Exon Skipping

6-well tissue culture plates were coated with Matrigel (BD Biosciences) (0.1 mg/ml) for 30 minutes at 37°C and then air dried in a tissue culture flow cabinet. Primary myoblasts from the index case were transfected by means of the nucleofection technique from Amaxa GmbH (Cologne, Germany) using the Nucleofector® Kit V, according to the manufacturer's protocol. In brief, 10^6^ myoblasts were trypsinized, washed in PBS and resuspended in 100 µl of solution V containing 300 nM of AVI-4658 ((+66+95 CTCCAACATCAAGGAAGATGGCATTTCTAG); AVI BioPharma). Immediately, cells were transferred into a cuvette and pulsed in a Nucleofector II device (Amaxa) using program B-32. Subsequently, the cells were diluted with 600 µL of prewarmed growth medium (37°C) and then distributed between the 6 wells of the plate each containing 1 mL of prewarmed medium. Following transfection the cells were left to recover overnight at 37°C and 5% CO_2_. The following day, medium was replaced with differentiation medium (DMEM containing 2% horse serum, penicillin-streptomycin (1 U/mL and 1 µg/mL, respectively), and 2 mM l-glutamine. After 3 days of differentiation, two wells from the 6-well plate were processed for RNA extraction and subsequent RT-PCR (detailed protocol follows), and the remaining four wells were kept in differentiation medium for another 7 days before analyzing dystrophin expression by western blot (protocol described in detail above).

RNA was extracted using TriZol (Invitrogen). Aliquots of 400 ng of total RNA were used for RT-PCR analysis (55°C for 35 min) in a 20 µl reaction using *C. therm* polymerase (Roche) a specific primer (4-1R; table S1). 3 µl of this reaction were used as template for a primary PCR performed by 20 cycles of 94°C (40 s), 60°C (40 s) and 72°C (80 s), with primers 4-1F and 4-1R (table S1). 1.5 µl of this reaction was later used as template for a nested PCR using primers 4-2F and 4-2R, consisting of 30 cycles of 94°C (40 s), 60°C (40 s) and 72°C (80 s). PCR products were analyzed on 1.5% agarose gels in Tris-acetate/EDTA (TAE) buffer, extracted and sequenced as detailed above.

## Supporting Information

Table S1 Table S1 Primer pairs used to amplify overlapping regions of cDNA from the index case (1-1 to 1-12 and 2-1 to 2-10), genomic DNA to demonstrate the mutation (3-1) and for the RFLP (3-2) and nested primer pairs for the exon skipping (4-1 and 4-2). For primer pair 4-2, a 666 bp product (in parentheses) is the size of the product expected with deletion of exon 50 due to the underlying mutation.
(0.04 MB DOC)
