# Supplementary material for: A Duchenne Muscular Dystrophy Gene Hot Spot Mutation in Dystrophin-Deficient Cavalier King Charles Spaniels Is Amenable to Exon 51 Skipping
Source: PLoS One. 2010 Jan 13;5(1):e8647. doi: 10.1371/journal.pone.0008647 (PMC2800183; doi:10.1371/journal.pone.0008647)
Supplement: Table S1 — Primer pairs used to amplify overlapping regions of cDNA from the index case (1-1 to 1-12 and 2-1 to 2-10), genomic DNA to demonstrate the mutation (3-1) and for the RFLP (3-2) and nested primer pairs for the exon skipping (4-1 and 4-2). For primer pair 4-2, a 666 bp product (in parentheses) is the size of the product expected with deletion of exon 50 due to the underlying mutation. (0.04 MB DOC) [file pone.0008647.s001.doc]

**Table S1**  Primer pairs used to amplify overlapping regions of cDNA from the index case (1-1 to 1-12 and 2-1 to 2-10), genomic DNA to demonstrate the mutation (3-1) and for the RFLP (3-2) and nested primer pairs for the exon skipping (4-1 and 4-2). For primer pair 4-2, a 666 bp product (in parentheses) is the size of the product expected with deletion of exon 50 due to the underlying mutation.

| Primer name | Forward (F) (5’-3’) | Reverse (R) (5’-3’) | Product size (bp) |
| --- | --- | --- | --- |
| 1-1 | ctctacagaatcctggcatc | cttcaatgctcacttgttga | 1000 |
| 1-2 | tgtcattaacttcaccacca | gttgtacttggcgttttagg | 1013 |
| 1-3 | aaatgactggctaaccaaaa | gttttcaaccagttttcagc | 1185 |
| 1-4 | caattgctaagcgagagact | ggtggtgagagtgtcaagtt | 1290 |
| 1-5 | ccttaaaaggaggtttggat | gttgctttctttctgtcacc | 1242 |
| 1-6 | attataatgagctgggagca | caaatttgctctcaatttcc | 1239 |
| 1-7 | aaaaattagccagcctacct | atttccttgatgtcaaatgg | 1390 |
| 1-8 | agcagcagctaaaagaaaaa | atctgtgtgagcttcgattt | 1502 |
| 1-9 | aacccacagattactgcaac | aaatgggctttacacagaga | 1575 |
| 1-10 | attggctgctgaatgtttat | attgtgtcctctctcattgg | 1516 |
| 1-11 | gaatggtacaacggtgtctt | taaagcaagaaagcaaaacc | 1553 |
| 1-12 | aaaatcacttccctctcctc | actgcgtgctttattgagat | 1304 |
| 2-1 | aagaatgaagcagaaccaga | aaattttcaagcgaatcaag | 590 |
| 2-2 | ctggcatgagttattgtcct | aacttcatggaaacatcctg | 573 |
| 2-3 | tattgctgacaaagtggatg | aatttcaagcatttttccaa | 528 |
| 2-4 | gaagtctgaagtggaaatgg | atccacattctggtcaaaag | 523 |
| 2-5 | ggaaatgttggtggaagata | tctcgctttctctcatctgt | 600 |
| 2-6 | acaaaaattgcttgaaccac | caaatttgctctcaatttcc | 521 |
| 2-7 | ctacctgaacccagagatga | tctgtcaaatctcccttgtc | 597 |
| 2-8 | aataaaaagacagcagcattg | ttccaggttcaagtggaata | 556 |
| 2-9 | agaggctagaggaacagaaga | atcagagctgagatcttcca | 596 |
| 2-10 | ccatttgacatcaaggaaat | gattgctggtcttgtttttc | 547 |
| 3-1 | taaacgtcatatccccatgt | ctcagtgttgtgcttttgaa | 635 |
| 3-2 | tagggtggttggctaaaata | atttccaaggagcctactct | 449 |
| 4-1 | tagaaatctgcctgagaagc | gcttggtttctgtgattttc | 935 |
| 4-2 | ccatttgacatcaaggaaat | ttttcttttggattgcatct | 775 (666) |
